# Chemical Profiling, Antifungal and Insecticidal Activities of *Santolina chamaecyparissus* L. Essential Oil: An Integrated Experimental and In Silico Study

**DOI:** 10.3390/cimb48101060

**Published:** 2026-10-14

**Authors:** Aziz Zouhri, Rafik El-Mernissi, Assia Houiat, Bahia Abdelfattah, Oussama Khibech, Yahya El-Mernissi, Mohamed Bouhrim, Joe Miantezila Basilua, Otmane Zouirech, Mohamed S. Alharbi, Ramzi A. Mothana, Hanan M. Al-Yousef, Nadia Senhaji, Lhoussain Hajji

**Affiliations:** 1 Bioactives and Environmental Health Laboratory, Faculty of Sciences, Moulay Ismail University, Meknes 11201, Morocco; 2 Biotechnology, Environment, Agri-Food and Health Laboratory, Faculty of Sciences Dhar El Mahraz, Sidi Mohamed Ben Abdellah University, Fez 30003, Morocco; 3 Laboratory of Physical Chemistry of Materials, Natural Substances and Environment (LAMSE), Chemistry Department, Faculty of Sciences and Techniques of Tangier, Tangier 90090, Morocco; 4 Laboratory of Applied Chemistry and Environment (LCAE), Faculty of Science, University Mohammed Premier, Oujda 60000, Morocco; 5 Research Unit in Applied Chemistry, Faculty of Science and Techniques, Abdelmalek Essaadi University, Al Hoceima 32003, Morocco; 6 Biological Engineering Laboratory, Faculty of Sciences and Techniques, Sultan Moulay Slimane University, Beni Mella 23000, Morocco; 7 Department of Biostatistics and Mathematics, Faculty of Pharmacy, Paris Cité University, 4, Avenue de l’Observatoire, 75006 Paris, France; 8 Laboratoire de Biotechnologie Conservation et Valorisation des Bioressources (LBCVB), Faculty of Sciences Dhar el Mehraz, Sidi Mohamed Ben Abdellah University, Fez 30000, Morocco; 9 Department of Pharmacognosy, College of Pharmacy, King Saud University, Riyadh 11451, Saudi Arabia

**Keywords:** *Santolina chamaecyparissus*, essential oil, GC–MS, antifungal activity, *Rhyzopertha dominica*, molecular docking, ADMET

## Abstract

*Santolina chamaecyparissus* L. essential oil (EO) was investigated for its chemical composition, antifungal and insecticidal activities, together with complementary molecular docking and ADMET analyses. GC–MS revealed a monoterpene-rich profile dominated by santolina triene (48.44%), α-pinene (14.05%), camphor (8.80%), myrcene (5.17%), and α-thujene (4.26%). The EO exhibited antifungal activity against *Fusarium oxysporum* and *Aspergillus brasiliensis*, with MIC and MFC values of 4 mg/mL and concentration-dependent inhibition of mycelial growth. Insecticidal activity against *Rhyzopertha dominica* was demonstrated through fumigant, topical-contact, and treated-grain assays, with efficacy varying according to concentration and exposure duration. Molecular docking identified α-thujene and p-cymene as the highest-ranked tested EO constituents against fungal CYP51B and insect AChE, respectively. ADMET predictions highlighted favorable physicochemical characteristics alongside potential toxicity-related liabilities requiring experimental verification. These findings provide a basis for further investigation of *S. chamaecyparissus* EO and its constituents as potential natural antifungal and insecticidal agents.

## 1. Introduction

Essential oils (EOs) are complex mixtures of volatile secondary metabolites, predominantly mono- and sesquiterpenes and, to a lesser extent, phenylpropanoids, that contribute to plant defense and ecological interactions. Their chemical diversity underlies a broad range of reported biological properties, including antimicrobial, antifungal, antioxidant, and insecticidal activities [1,2]. These properties have stimulated interest in EOs as potential sources of bioactive compounds for pharmaceutical, agri-food, and crop-protection applications. In particular, their capacity to affect multiple cellular and physiological targets, including microbial membrane integrity and insect neurological processes, provides a rationale for investigating them as complementary sources of antifungal and insecticidal agents [3,4]. Nevertheless, their biological efficacy, toxicity, stability, and environmental safety are strongly dependent on chemical composition and exposure conditions and therefore require compound- and application-specific evaluation.

Fungal pathogens represent a major challenge in both agriculture and human health. Phytopathogenic fungi such as *Fusarium oxysporum* are responsible for economically important plant diseases, whereas species of *Aspergillus* include opportunistic pathogens of clinical relevance [5]. The increasing concern surrounding antifungal resistance further emphasizes the need to identify structurally diverse bioactive compounds and explore alternative or complementary antifungal strategies. In particular, sterol 14α-demethylase (CYP51), a cytochrome P450 enzyme involved in ergosterol biosynthesis, represents an established molecular target of azole antifungals [6]. The identification of natural compounds capable of interacting with fungal sterol-biosynthesis pathways may therefore provide useful leads for subsequent mechanistic and antifungal investigations.

In parallel, stored-product insects cause substantial quantitative and qualitative losses in cereals and other food commodities during storage. *Rhyzopertha dominica* (F.) (Coleoptera: Bostrichidae), commonly known as the lesser grain borer, is an important pest of stored cereals because both adults and larvae damage kernels and can markedly reduce grain quality [7]. Although synthetic insecticides and fumigants remain important tools for controlling stored-product pests, their intensive or inappropriate use may contribute to insecticide resistance, chemical residues, and adverse effects on non-target organisms [8]. Consequently, plant-derived products, including EOs, have received increasing attention as potential sources of insecticidal compounds. Their activity may occur through different exposure routes, including fumigant, contact, and ingestion-associated effects, making the evaluation of multiple exposure pathways particularly relevant when assessing their potential for stored-product protection.

Within this context, *Santolina chamaecyparissus* L. (Asteraceae), commonly known as lavender cotton, is an aromatic perennial species characteristic of the Mediterranean region [9]. The plant has traditionally been used for several purposes associated with its aromatic and medicinal properties, and its EO contains numerous volatile terpenoids. Previous investigations have shown that the chemical composition of *S. chamaecyparissus* EO can vary considerably according to geographical origin, environmental conditions, phenological stage, and other factors influencing secondary metabolism [9,10,11,12]. Such chemical variability is particularly important because differences in the relative abundance of volatile constituents may substantially influence the biological properties of an EO.

Despite previous reports on the chemical composition and selected biological properties of *S. chamaecyparissus* EO, important questions remain regarding the relationship between its volatile profile and its antifungal and insecticidal activities. In particular, information on its activity against *F. oxysporum* and *Aspergillus brasiliensis*, together with a systematic assessment of its insecticidal effects against *R. dominica* through complementary exposure routes, remains limited. Furthermore, experimental observations of whole-EO activity do not by themselves identify the molecular targets potentially involved in these effects. Integrating chemical characterization and biological assays with target-based computational approaches may therefore provide additional hypotheses regarding the contribution of individual EO constituents to the observed activities.

Accordingly, molecular docking can be used as a complementary approach to examine whether selected EO constituents can be accommodated within biologically relevant target-binding sites. Fungal sterol 14α-demethylase (CYP51B) is particularly relevant to antifungal activity because of its central role in ergosterol biosynthesis, whereas acetylcholinesterase (AChE), which regulates cholinergic neurotransmission, represents a biologically relevant target for investigating potential insect neurotoxicity. However, docking scores and predicted interactions should be interpreted as mechanistic hypotheses rather than direct evidence of enzyme inhibition. Similarly, in silico absorption, distribution, metabolism, excretion, and toxicity (ADMET) predictions can provide preliminary information for prioritizing compounds and identifying potential liabilities that require subsequent experimental verification.

Based on these considerations, we hypothesized that the monoterpene-rich EO of *S. chamaecyparissus* collected in northern Morocco exhibits antifungal and insecticidal activities and that selected constituents may show plausible interactions with molecular targets relevant to these effects. Therefore, the present study aimed to characterize the chemical composition of *S. chamaecyparissus* EO by GC–MS; evaluate its antifungal activity against *F. oxysporum* and *A. brasiliensis* using minimum inhibitory concentration (MIC), minimum fungicidal concentration (MFC), and mycelial growth inhibition assays; assess its insecticidal activity against *R. dominica* through fumigant, topical-contact, and treated-grain exposure assays; and explore potential molecular interactions of selected EO constituents with CYP51B and AChE through molecular docking, complemented by ADMET prediction of prioritized compounds. This integrated experimental and computational framework was designed to establish a chemical and biological profile of *S. chamaecyparissus* EO and to generate mechanistic hypotheses for its observed antifungal and insecticidal effects.

## 2. Materials and Methods

### 2.1. Plant Material and Essential Oil Preparation

The aerial parts of *Santolina chamaecyparissus* L. were collected in April 2023 from the Taounate region, northern Morocco. The plant material was botanically authenticated by Amina Bari, botanist, and a voucher specimen (No. 034S6/26) was deposited in the herbarium of the Department of Biology, Faculty of Sciences Dhar El Mahraz, Sidi Mohamed Ben Abdellah University, Fez, Morocco.

Essential oil (EO) was obtained by hydrodistillation using a Clevenger-type apparatus. Briefly, 200 g of aerial plant material were subjected to hydrodistillation for 4 h. Following distillation, the EO was separated from the aqueous phase and dried over anhydrous sodium sulfate to remove residual moisture. The resulting oil was transferred into airtight amber vials and stored at 4 °C until chemical and biological analyses. The EO yield was determined by calculating the percentage (*v*/*w*) based on the weight of the dried plant material.

### 2.2. Essential Oil Chemical Characterization by GC–MS

The chemical composition of *Santolina chamaecyparissus* L. essential oil was analyzed by gas chromatography coupled with mass spectrometry (GC–MS) using a Thermo Scientific TRACE GC ULTRA system equipped with a mass-selective detector. Separation was performed on a TG-1MS capillary column (30 m × 0.25 mm; film thickness, 0.25 μm) using helium as the carrier gas at a constant flow rate of 1.5 mL/min. The injector and interface temperatures were maintained at 250 °C. The oven temperature was initially set at 90 °C and subsequently increased at 3.5 °C/min to 250 °C, which was maintained for 3 min.

Prior to analysis, 1 μL of EO was diluted in 1 mL of cyclohexane, and a 1 μL aliquot of the resulting solution was injected using a TriPlus RSH autosampler. Mass spectra were acquired in electron-ionization (EI) mode at 70 eV over an *m*/*z* range of 50–550. The volatile constituents were tentatively identified by comparison of their mass spectra with those available in the NIST mass-spectral library. Relative abundances were estimated from the corresponding chromatographic peak areas and expressed as percentages of the total detected peak area.

### 2.3. Antifungal Activity

#### 2.3.1. Fungal Strains and Culture Conditions

The antifungal activity of *S. chamaecyparissus* EO was evaluated against *Fusarium oxysporum* (ATCC 48112) and *Aspergillus brasiliensis* (ATCC 16404). The fungal strains were provided by the Laboratory of Biotechnology, Environment, Agri-Food, and Health, Faculty of Sciences Dhar El Mahraz, Sidi Mohamed Ben Abdellah University, Fez, Morocco.

#### 2.3.2. Preparation of Essential-Oil Solutions and Fungal Inocula

A stock solution of the EO was prepared at 100 mg/mL using Tween 80 (0.1%, *v*/*v*) as the dispersing agent and homogenized thoroughly. Serial dilutions were subsequently prepared using the same diluent to obtain the concentrations required for the antifungal assays.

Conidia were harvested from 7-day-old fungal cultures grown on potato dextrose agar (PDA) and suspended in sterile 0.9% NaCl solution. Conidial density was determined using a Malassez counting chamber and adjusted to approximately 10^6^ conidia/mL [13].

#### 2.3.3. Minimum Inhibitory and Fungicidal Concentrations and Mycelial Growth Inhibition

The antifungal activity of the EO was evaluated according to the method described by Oukhouia et al. [14], with appropriate adaptation. Sterile PDA medium was cooled to approximately 45 °C and supplemented with EO to obtain final concentrations of 0, 0.5, 1, 2, 4, and 6 mg/mL. The supplemented media were poured into sterile Petri dishes (90 × 16 mm). Each plate was inoculated with 15 μL of a fungal suspension containing approximately 10^6^ conidia/mL and incubated at 27 °C for 7 days.

Antifungal activity was assessed by measuring radial mycelial growth. Mycelial growth inhibition (MGI) was calculated relative to the untreated control according to the method described by Pandey et al. [15], where *D*_(c)_ represents the mean colony diameter in the control and *D*_(t)_ represents the mean colony diameter in the EO-treated plates.

The minimum inhibitory concentration (MIC) was defined as the lowest EO concentration at which no visible fungal growth was observed. The minimum fungicidal concentration (MFC) was determined according to the method described by Oshim et al. [16] with modifications. Briefly, agar plugs approximately 6 mm in diameter were aseptically removed from plates corresponding to EO concentrations showing no visible fungal growth at the end of the incubation period. The plugs were transferred, mycelium-side down, onto fresh Sabouraud dextrose agar plates without the tested antifungal agent and incubated at 27 °C for 3 days. The MFC was defined as the lowest EO concentration for which no visible fungal regrowth was observed after transfer to the antifungal-agent-free medium. The Tween 80 solvent control was processed under the same conditions and showed no detectable antifungal activity. All experiments were performed in triplicate.

### 2.4. Insecticidal Activity Against Rhyzopertha dominica

#### 2.4.1. Insect Rearing and Maintenance

Adult *Rhyzopertha dominica* were obtained from infested wheat supplied by the National Seed Marketing Company (SONACOS S.A., Rabat, Morocco). The insects were reared in 1 L glass containers containing 250 g of wheat as a food substrate. Fifty adults were introduced into each container, which was subsequently covered with muslin to ensure adequate ventilation. The cultures were maintained at 30 °C and 60–70% relative humidity (RH) under a 16:8 h light:dark photoperiod. Unsexed adults aged 2–7 days were used for the insecticidal bioassays.

#### 2.4.2. Fumigant Toxicity Assay

The fumigant toxicity of *S. chamaecyparissus* EO against adult *R. dominica* was evaluated according to the method described by Shahinfar et al. [17], with appropriate adaptation. The assay was conducted in 100 mL glass bottles, each containing 20 unsexed adults (2–7 days old) and 5 g of corn as a food source. Whatman No. 2 filter-paper discs (2.5 cm in diameter) were attached beneath the bottle lids and treated with EO preparations corresponding to concentrations of 0, 3, 6, and 12 μL/L of air. The control treatment consisted of 0.1% (*v*/*v*) Tween 80.

The bottles were maintained at 30 °C and 60–70% RH under a 16:8 h light:dark photoperiod for 4 days. Mortality was recorded every 24 h. Insects showing no movement of the body, head, or antennae were considered dead [18]. Each treatment was performed in triplicate, and mortality was corrected using Abbott’s formula [19].

#### 2.4.3. Topical Contact Toxicity Assay

The topical toxicity of *S. chamaecyparissus* EO against adult *R. dominica* was evaluated according to the method described by Plata-Rueda et al. [20], with appropriate adaptation. The EO was diluted in 0.1% (*v*/*v*) Tween 80 to obtain the tested doses (0, 0.05, 0.10, and 0.20 μL/insect). A 0.2-μL aliquot of the corresponding preparation was applied topically to the thoracic region of each adult using a micropipette. Twenty unsexed adults were used per replicate.

Following treatment, the insects were transferred to Petri dishes and maintained at 30 °C and 60–70% RH under a 16:8 h light:dark photoperiod. Mortality was assessed after 24 h. Three replicates were performed for each treatment, and corrected mortality was calculated using Abbott’s formula [19].

#### 2.4.4. Contact Toxicity on Treated Grains

The toxicity of the EO applied to wheat grains was evaluated following the method [21], with appropriate adaptation. Fifty grams of wheat grains were placed in 100 mL glass vials and treated with EO at concentrations of 0, 0.03, 0.05, and 0.10% (*w*/*v*). The grains were mixed for 1 min to promote uniform distribution of the EO and subsequently allowed to dry for 10 min. Twenty unsexed adult *R. dominica* were then introduced into each vial.

The vials were covered with muslin to allow ventilation and maintained at 30 °C and 60–70% RH under a 16:8 h light:dark photoperiod for 6 days. Mortality was recorded daily; dead insects were removed after each observation. Each treatment was performed in triplicate, and corrected mortality was calculated using Abbott’s formula [19].

### 2.5. Molecular Docking Studies

**Figure 1:**
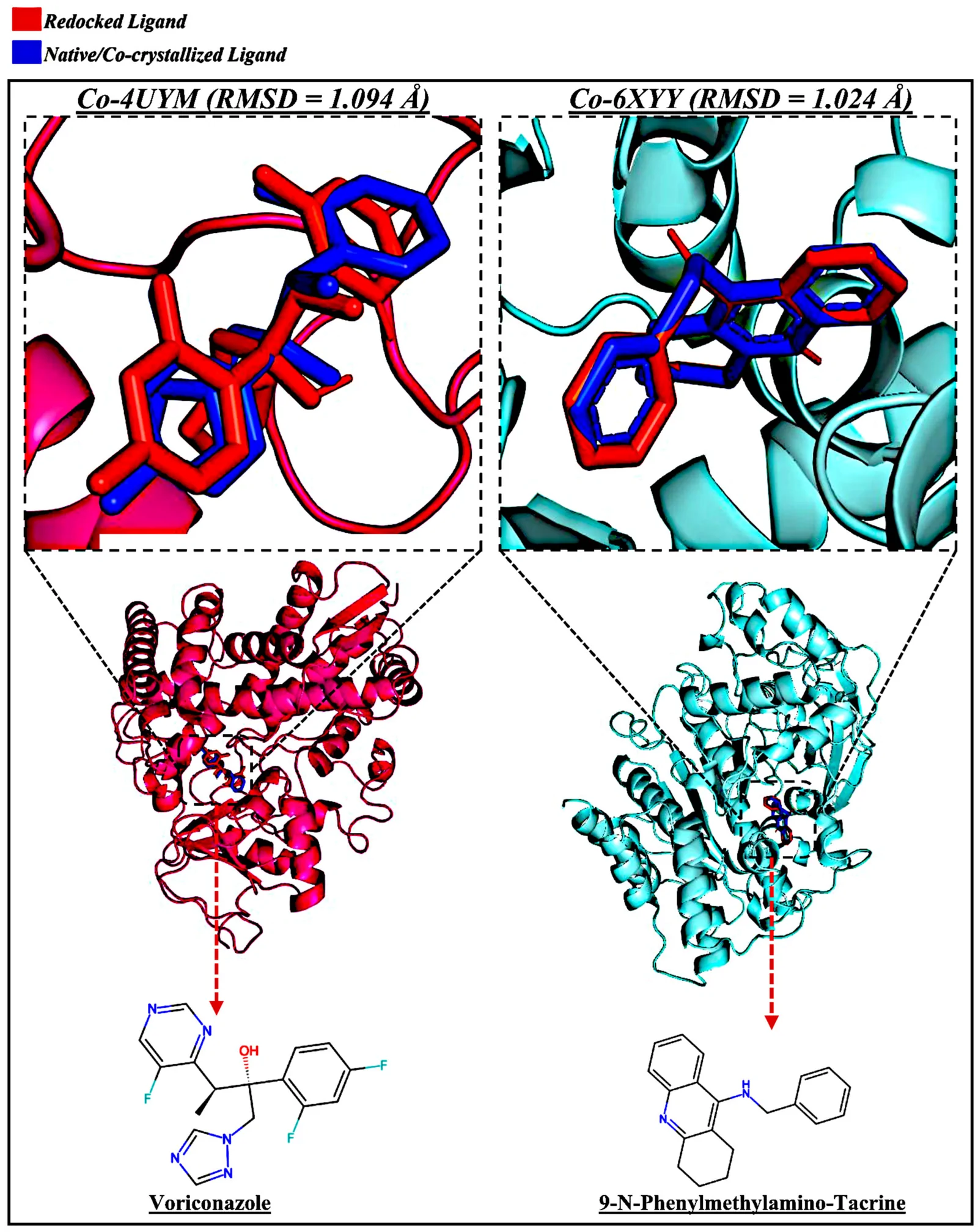
Validation of the docking protocol by re-docking the co-crystallized ligands voriconazole (Co-4UYM) and 9-N-phenylmethylamino-tacrine (Co-6XYY; PDB ligand code 760) into CYP51B (PDB ID: 4UYM) and acetylcholinesterase (PDB ID: 6XYY), respectively. The re-docked poses (red) were superimposed on the crystallographic conformations (blue), yielding RMSD values of 1.094 and 1.024 Å, respectively. The chemical structures of both ligands are shown below their corresponding complexes.

Computational molecular docking was performed using AutoDock Vina v1.2.0 through the PyRx v0.9 interface [22,23,24]. The X-ray structures of *Aspergillus fumigatus* sterol 14-alpha-demethylase CYP51B in complex with voriconazole (PDB ID: 4UYM; resolution 2.55 Å) and *Drosophila melanogaster* acetylcholinesterase in complex with tacrine derivative 760 (PDB ID: 6XYY; resolution 2.70 Å) were retrieved from the RCSB Protein Data Bank. Receptors were prepared in AutoDockTools v1.5.7 by removing crystallographic water molecules and non-essential heteroatoms, adding polar hydrogens, assigning the charges required for PDBQT generation, and deleting the co-crystallized ligands before docking. The heme prosthetic group of CYP51B was retained to preserve the catalytic architecture of the azole-binding site. The eight essential-oil constituents selected for computational evaluation α-thujene, p-cymene, camphene, camphor, isoborneol, isopulegol, santolina triene, and myrcene were energy-minimized in PyRx, converted to PDBQT format, and treated as flexible ligands with appropriate torsion definitions, whereas the receptors were kept rigid. The search exhaustiveness was set to 10. For 4UYM, the grid was centered at x = 134.91, y = 196.73, and z = 3.71 Å, with dimensions x = 14.98, y = 14.50, and z = 12.39 Å. For 6XYY, the grid was centered at x = 32.72, y = 67.61, and z = 9.32 Å, with dimensions x = 12.26, y = 11.13, and z = 7.41 Å. Docking reliability was assessed by re-docking each native ligand into its crystallographic binding pocket. Superposition of the predicted and experimental poses yielded RMSD values of 1.094 Å for Co-4UYM and 1.024 Å for Co-6XYY (Figure 1), both below the commonly applied 2.0 Å pose-reproduction threshold and therefore supportive of the protocol for comparative pose prediction and interaction analysis. For each ligand-target pair, the most favorable Vina pose was retained for ranking and visualization. Molecular visualization and figure preparation were carried out using PyMOL v2.5 and BIOVIA Discovery Studio 2024.

### 2.6. ADMET Prediction

Canonical SMILES strings for the two highest-ranked constituents, alpha-thujene and p-cymene, were retrieved from PubChem and submitted individually to the admetSAR3.0 platform. The analysis generated basic physicochemical descriptors and medicinal-chemistry filters, together with predictions covering absorption, distribution, metabolism, excretion, human-organ toxicity, general toxicity endpoints, and endocrine-disruption endpoints. The numerical outputs were summarized using the platform’s radar charts, basic-property displays, and min-max normalized heat maps [25]. All outputs were treated as structure-based model predictions for prioritization and safety-hypothesis generation; they were not interpreted as substitutes for experimental pharmacokinetic, toxicological, or environmental-safety measurements.

### 2.7. Statistical Analysis

All experimental data are expressed as mean ± standard deviation (SD) of three independent replicates. Statistical analyses were performed using GraphPad Prism version 9.5. Differences among treatments were evaluated by two-way analysis of variance (ANOVA), followed by Tukey’s multiple-comparison test, where appropriate. Differences were considered statistically significant at *p* < 0.05.

## 3. Results and Discussion

### 3.1. Chemical Composition of Santolina chamaecyparissus Essential Oil

**Figure 2:**
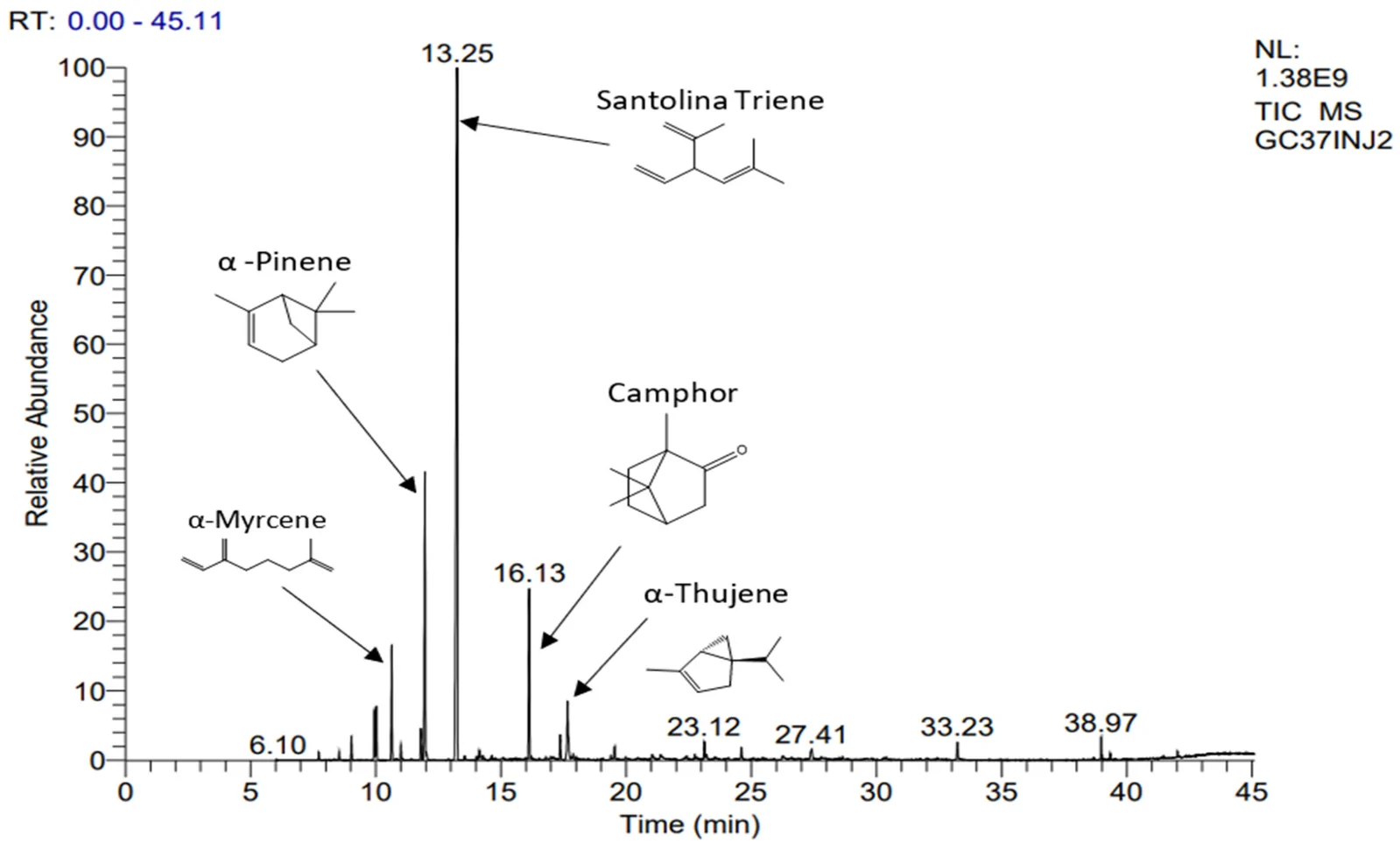
GC–MS total ion chromatogram of *Santolina chamaecyparissus* L. essential oil. The major peaks corresponding to santolina triene, α-pinene, camphor, myrcene, and α-thujene are labelled directly on the chromatogram.

The essential oil (EO) yield obtained from the aerial parts of *Santolina chamaecyparissus* was 0.21 ± 0.07% (*v*/*w*). GC–MS analysis revealed a volatile profile predominantly composed of monoterpenoid constituents (Table 1 and Figure 2). Based on mass-spectral library matching, santolina triene was the most abundant tentatively identified constituent, accounting for 48.44% of the total chromatographic area. Other quantitatively important constituents included α-pinene (14.05%), camphor (8.80%), myrcene (5.17%), and α-thujene (4.26%). Several additional constituents occurred at comparatively lower relative abundances. Overall, these results indicate a chemically heterogeneous but strongly monoterpene-dominated volatile fraction, with santolina triene representing the predominant component under the investigated growing and collection conditions.

The predominance of monoterpenes is consistent with the general volatile chemistry reported for *Santolina* species; however, the relative abundance of individual constituents can vary substantially among EO samples. Such variability may reflect differences in geographical origin, genotype or chemotype, phenological stage, environmental conditions, plant organ, harvesting period, and extraction procedure [26,27].

From a biological perspective, the observed activities should not be attributed exclusively to the predominant constituent. Although santolina triene represented nearly half of the chromatographic profile, the EO also contained several other monoterpenes that may contribute individually or collectively to its biological effects. Essential oils function as multicomponent mixtures, and their activity may result from additive, synergistic, or antagonistic interactions among constituents. This interpretation is also consistent with the subsequent molecular-docking results, in which the most abundant constituent was not necessarily the highest-ranked compound against the investigated molecular targets.

### 3.2. Fungal Activity of the Essential Oil

#### 3.2.1. Minimum Inhibitory and Fungicidal Concentrations

The antifungal activity of *S. chamaecyparissus* EO was evaluated against *A. brasiliensis* and *F. oxysporum*. As shown in Table 2, the EO inhibited visible fungal growth at a minimum inhibitory concentration (MIC) of 4 mg/mL for both strains. The corresponding minimum fungicidal concentration (MFC) was also 4 mg/mL, indicating that, under the conditions of the present assay, the concentration required to prevent visible growth coincided with that associated with fungicidal activity.

The comparable MIC and MFC values obtained for the two fungal species suggest a broadly similar susceptibility to the EO under the experimental conditions used. Nevertheless, these values should be interpreted in the context of the assay methodology, inoculum density, growth medium, incubation conditions, and chemical composition of the EO, all of which can substantially influence antifungal endpoints.

Previous investigations nevertheless support the antifungal potential of *S. chamaecyparissus*. Essential oils obtained from Tunisian *S. chamaecyparissus*, for example, inhibited several fungal species, including *Aspergillus fumigatus*, although the magnitude of activity varied according to the plant organ and fungal strain tested [11]. Other studies have also reported antifungal effects of *S. chamaecyparissus* preparations against *Candida* and *Fusarium* species. These findings support the antifungal potential of the species while simultaneously highlighting the substantial influence of chemical profile, extraction procedure, and experimental model on the measured activity [10,12].

#### 3.2.2. Inhibition of Mycelial Growth

**Figure 3:**
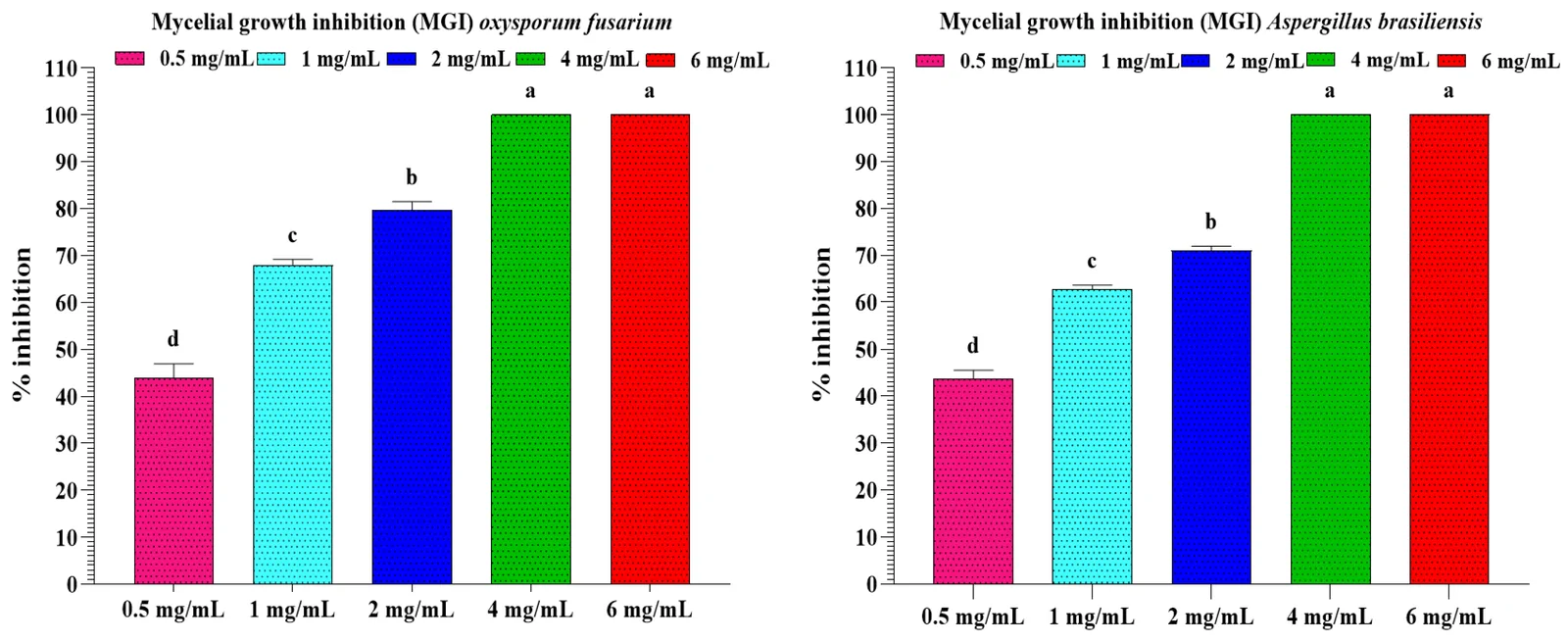
Mycelial growth inhibition (MGI) of the tested fungal strains by *Santolina chamaecyparissus* L. essential oil in agar medium. Data are expressed as means ± standard deviation (SD) (n = 3). Different letters (a, b, c, d) indicate statistically significant differences between treatments (*p* < 0.05).

In addition to the MIC/MFC endpoints, the effect of *S. chamaecyparissus* EO on fungal development was examined by monitoring mycelial growth across increasing EO concentrations (Figure 3). The EO produced a concentration-dependent reduction in mycelial growth in both *F. oxysporum* and *A. brasiliensis*, with the strongest inhibition observed at the highest tested concentrations. The progressive reduction in colony development with increasing EO concentration supports a dose-dependent antifungal effect rather than an isolated response at the MIC.

Monoterpenes are frequently associated with antifungal effects, particularly through interactions with hydrophobic cellular structures [28,29,30].

The observed inhibition of *F. oxysporum* is particularly relevant from an agricultural perspective. Previous work using *S. chamaecyparissus* preparations has also demonstrated inhibition of *Fusarium* species, although differences in extraction method and experimental conditions prevent direct comparison of potency [31]. For example, an aqueous *S. chamaecyparissus* extract showed moderate inhibition of *F. oxysporum* f. sp. *lentis*, whereas recent work with *S. chamaecyparissus* flower extracts reported concentration-dependent antifungal activity against *Neocosmospora* species [31,32]. Taken together, the present findings extend the available evidence by demonstrating antifungal activity of the investigated EO against both *F. oxysporum* and *A. brasiliensis* under the conditions used in this study.

### 3.3. Insecticidal Activity Against Rhyzopertha dominica

#### 3.3.1. Fumigant Toxicity

**Figure 4:**
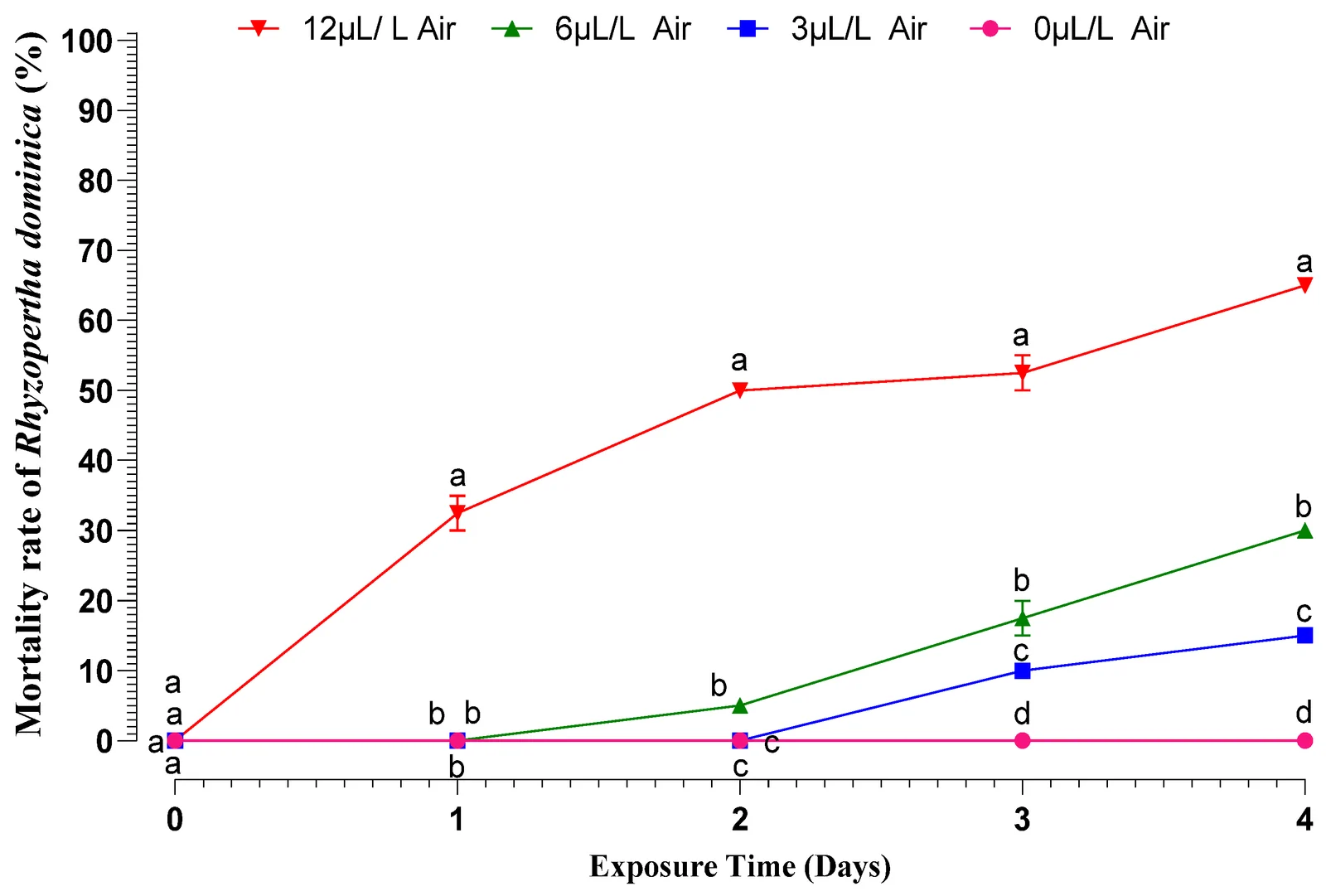
Fumigant activity of *Santolina chamaecyparissus* L. essential oil against *Rhyzopertha dominica*. Data are expressed as means ± standard deviation (SD) (n = 3). Different letters (a, b, c, d) indicate statistically significant differences between treatments at the same day (*p* < 0.05).

The fumigant activity of *S. chamaecyparissus* EO against adult *R. dominica* showed a clear concentration- and time-dependent pattern (Figure 4). At the highest tested concentration (12 μL/L air), mortality increased rapidly to approximately 30% after 24 h and 50% after 48 h, before reaching approximately 65% after 96 h. In contrast, 6 μL/L produced little mortality during the first 24 h, followed by a progressive increase to approximately 30% at 96 h. The 3 μL/L treatment exhibited still weaker activity, reaching only approximately 15% mortality at the end of the experiment, whereas no mortality was observed in the control. The statistical differences among concentrations, particularly at the later exposure periods, confirm that fumigant toxicity was strongly dependent on EO concentration.

The rapid response observed at 12 μL/L indicates that sufficiently high vapor concentrations can exert an acute toxic effect, whereas the delayed mortality observed at 3 and 6 μL/L suggests that lower vapor concentrations require prolonged exposure to produce measurable toxicity. Thus, the fumigant effect appears to depend on both the amount of volatile compounds present in the headspace and the duration of insect exposure.

This activity is consistent with the highly volatile and predominantly monoterpenic character of the EO. Santolina triene constituted the largest proportion of the chromatographic profile (48.44%), accompanied by α-pinene, camphor, myrcene, α-thujene, and other monoterpenes. Such volatile constituents can readily partition into the headspace of closed experimental systems, making fumigation a biologically plausible route of exposure. The individual contributions of these constituents and their possible interactions were not resolved experimentally.

#### 3.3.2. Topical Contact Toxicity

**Figure 5:**
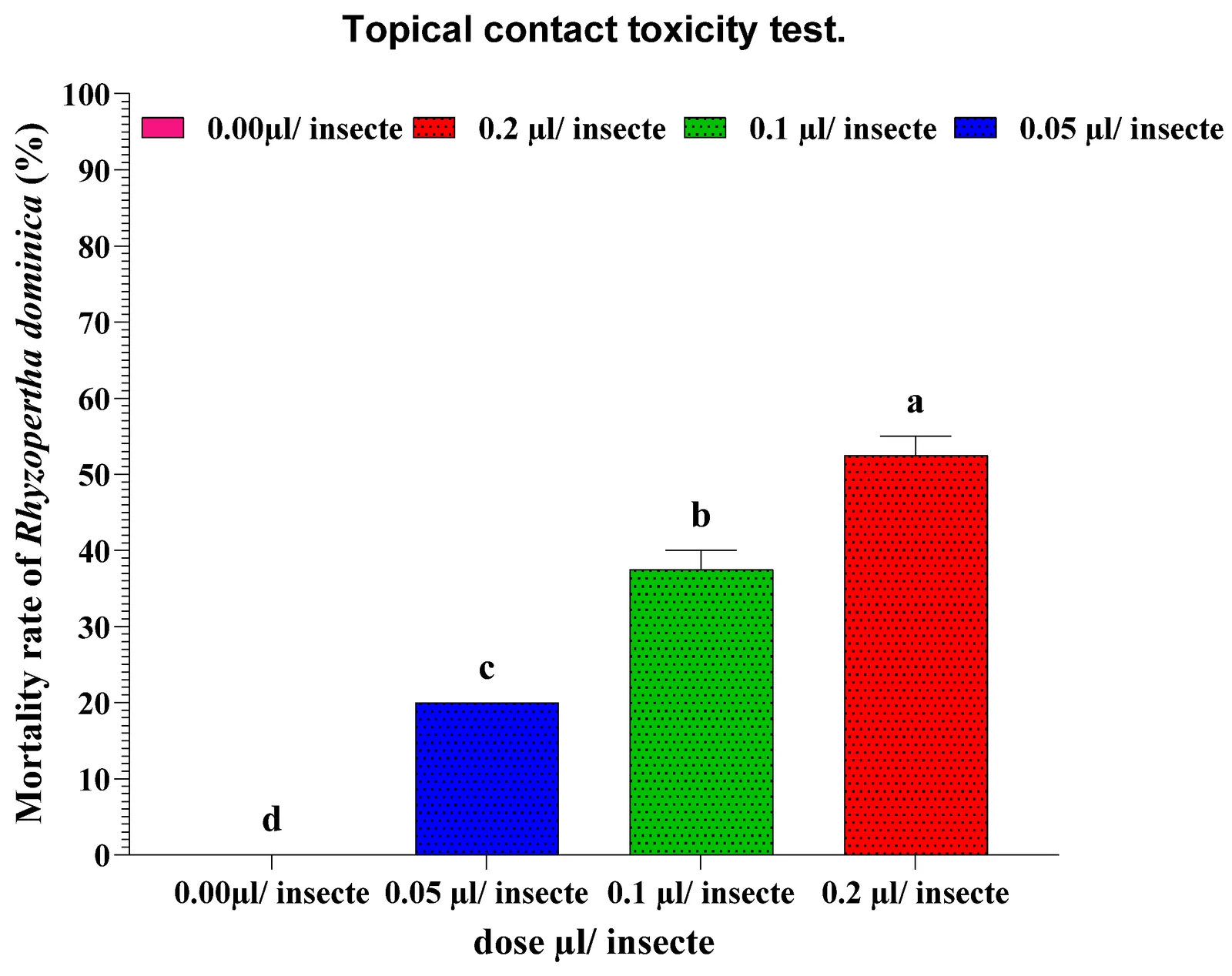
Topical contact toxicity of *Santolina chamaecyparissus* L. essential oil at different concentrations against adult *Rhyzopertha dominica* after 24 h of exposure. Data are expressed as means ± standard deviation (SD) (n = 3). Different letters (a, b, c, d) indicate significant differences among treatments (*p* < 0.05).

Topical application of *S. chamaecyparissus* EO produced a significant dose-dependent toxic effect against adult *R. dominica* after 24 h (Figure 5). Mortality increased from approximately 20% at 0.05 μL/insect to approximately 37% at 0.10 μL/insect and 52% at 0.20 μL/insect, whereas mortality was negligible in the untreated control. The different statistical groups assigned to the tested doses (*p* < 0.05) indicate that increasing the amount of EO applied directly to the insect significantly enhanced mortality.

The approximately 2.6-fold increase in mortality between 0.05 and 0.20 μL/insect demonstrates a marked dose–response relationship. Moreover, because this response occurred within only 24 h, direct contact appears to provide relatively rapid delivery of toxic constituents compared with the lower fumigant concentrations, which required longer exposure periods to produce substantial mortality. The lipophilic nature of the monoterpenes detected in the EO may favor their interaction with the insect cuticle and subsequent penetration into internal tissues. Nevertheless, cuticular penetration and specific physiological targets were not directly measured in the present study, and this interpretation should therefore be regarded as a plausible explanation rather than a demonstrated mechanism.

Importantly, the topical assay demonstrates that the insecticidal effect cannot be explained solely by accumulation of EO vapors in a closed environment. Direct exposure of the insect body to the EO was sufficient to induce mortality, indicating that *S. chamaecyparissus* EO can exert toxicity through more than one exposure route.

#### 3.3.3. Toxicity Through Treated Grains

**Figure 6:**
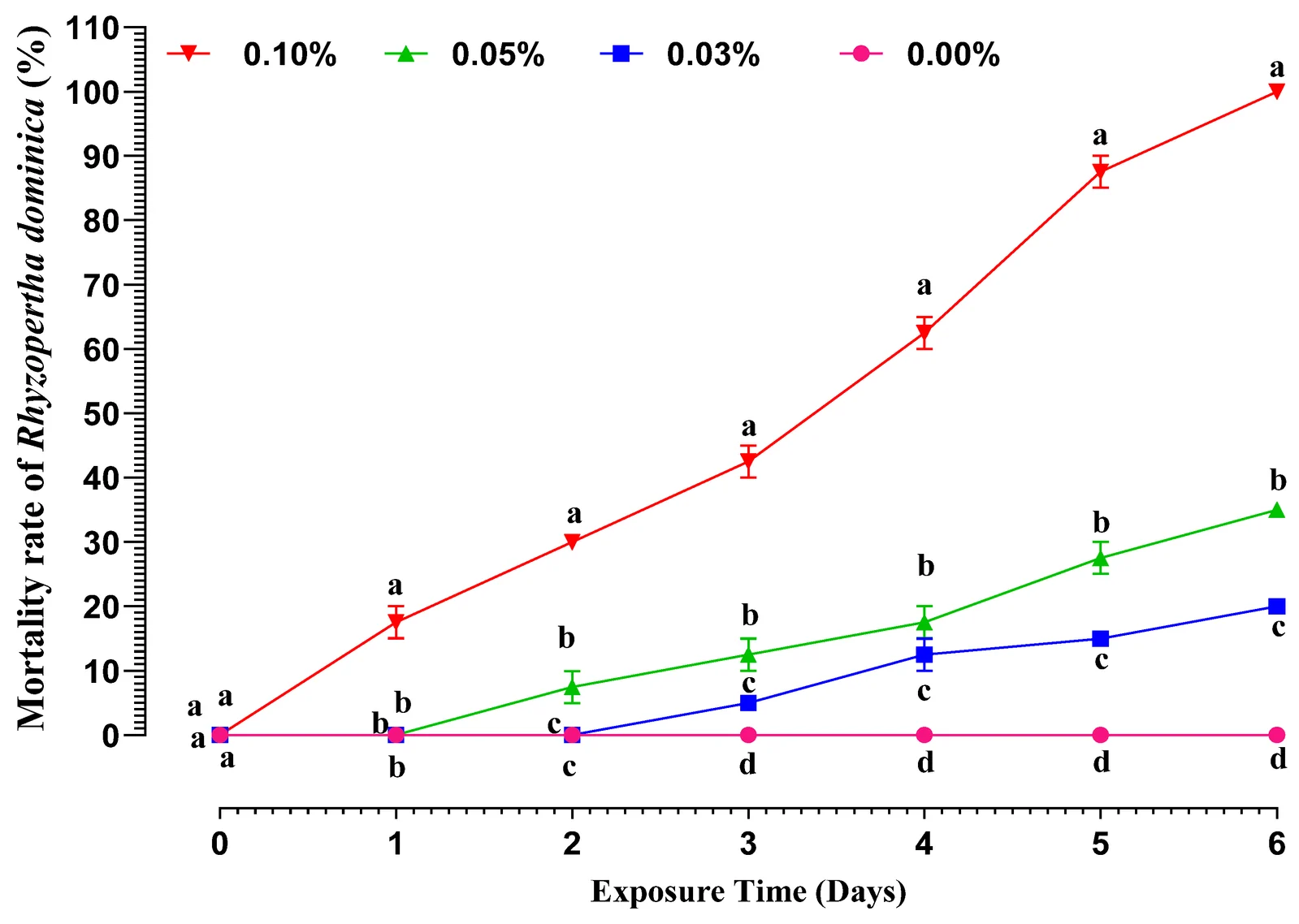
Mortality of adult *Rhyzopertha dominica* exposed to grains treated with *Santolina chamaecyparissus* L. essential oil at different concentrations after 144 h of exposure. Data are expressed as means ± standard deviation (SD) (n = 3). Different letters (a, b, c, d) indicate significant differences among treatments (*p* ≤ 0.05).

The treated-grain assay produced the strongest insecticidal response observed in the present study (Figure 6). Mortality was both concentration- and time-dependent. At 0.10% EO, mortality increased progressively from approximately 18% after 24 h to 30% after 48 h, 43% after 72 h, 63% after 96 h, and approximately 88% after 120 h, ultimately reaching 100% after 144 h. By comparison, the 0.05% treatment produced a slower increase in mortality, reaching approximately 35% after 144 h, while 0.03% EO resulted in approximately 20% mortality. No mortality was observed in the untreated control. The significant differences among treatments confirm the strong influence of EO concentration on insect mortality.

The complete mortality obtained with 0.10% EO after 144 h is particularly noteworthy because it demonstrates that the insecticidal activity was maintained when the EO was applied to the actual feeding substrate rather than delivered exclusively by direct topical application or fumigation. Furthermore, mortality continued to accumulate throughout the six-day observation period, suggesting that prolonged exposure to treated grains considerably enhances efficacy. The substantial difference between 0.10% and the two lower concentrations also indicates the existence of a pronounced concentration threshold under the experimental conditions.

Several exposure pathways may have contributed to this response. Adults remained continuously in contact with the treated wheat, allowing prolonged cuticular exposure, while volatile constituents released from the grain surface could simultaneously contribute a fumigant component. Feeding on treated grains may provide an additional route of exposure. Because these pathways were not experimentally separated, the exceptionally high mortality observed at 0.10% should be interpreted as the combined outcome of exposure to EO-treated grain rather than being attributed exclusively to contact, fumigation, or ingestion.

Comparison of the three bioassays reveals that the insecticidal efficacy of *S. chamaecyparissus* EO was strongly dependent on both the mode and duration of exposure. Fumigation produced substantial but incomplete mortality, reaching approximately 65% at 12 μL/L air after 96 h, whereas direct topical application resulted in approximately 52% mortality at 0.20 μL/insect within 24 h. In contrast, continuous exposure to grains treated with 0.10% EO resulted in complete mortality after 144 h. Although direct quantitative comparison among these assays must be made cautiously because the doses are expressed in different units and the exposure periods differ, the results collectively demonstrate that prolonged exposure through the treated substrate generated the highest final mortality under the tested conditions.

This distinction is biologically meaningful. The moderate fumigant effect suggests that volatility contributes to toxicity but may not fully account for the activity of the EO, while the topical assay confirms that direct contact can also produce rapid mortality. The substantially greater cumulative mortality on treated grains may therefore reflect prolonged and potentially multiple routes of exposure. Such multi-route activity could be advantageous in stored-grain protection, where insects may simultaneously encounter EO residues on grain surfaces and volatile compounds within the storage atmosphere [33,34].

The chemical complexity of the EO should also be considered when interpreting these effects. Santolina triene represented 48.44% of the chromatographic profile, yet abundance alone does not demonstrate biological importance. α-Pinene, camphor, myrcene, α-thujene, and other minor constituents may also contribute to the observed toxicity, either independently or through interactions within the EO mixture [29,35].

### 3.4. Molecular Docking

Structure-based docking was used as a mechanistic bridge between the chemical composition of the essential oil and the antifungal and insecticidal phenotypes observed experimentally. CYP51B was selected because sterol 14-alpha-demethylation is central to fungal membrane-sterol biosynthesis and the 4UYM structure contains the established azole inhibitor voriconazole, whereas insect AChE was selected because disruption of acetylcholine hydrolysis provides a biologically plausible route to neurotoxicity and mortality, and 6XYY offers a structurally defined insect AChE gorge bound to a tacrine derivative [36]. Importantly, the experimental organisms were *Aspergillus brasiliensis*, *Fusarium oxysporum*, and *Rhyzopertha dominica*, whereas the structural models originated from *A. fumigatus* and *D. melanogaster*. Accordingly, these receptors were used as homologous mechanistic proxies for target engagement rather than as species-identical confirmation of the biological mechanism.

**Figure 7:**
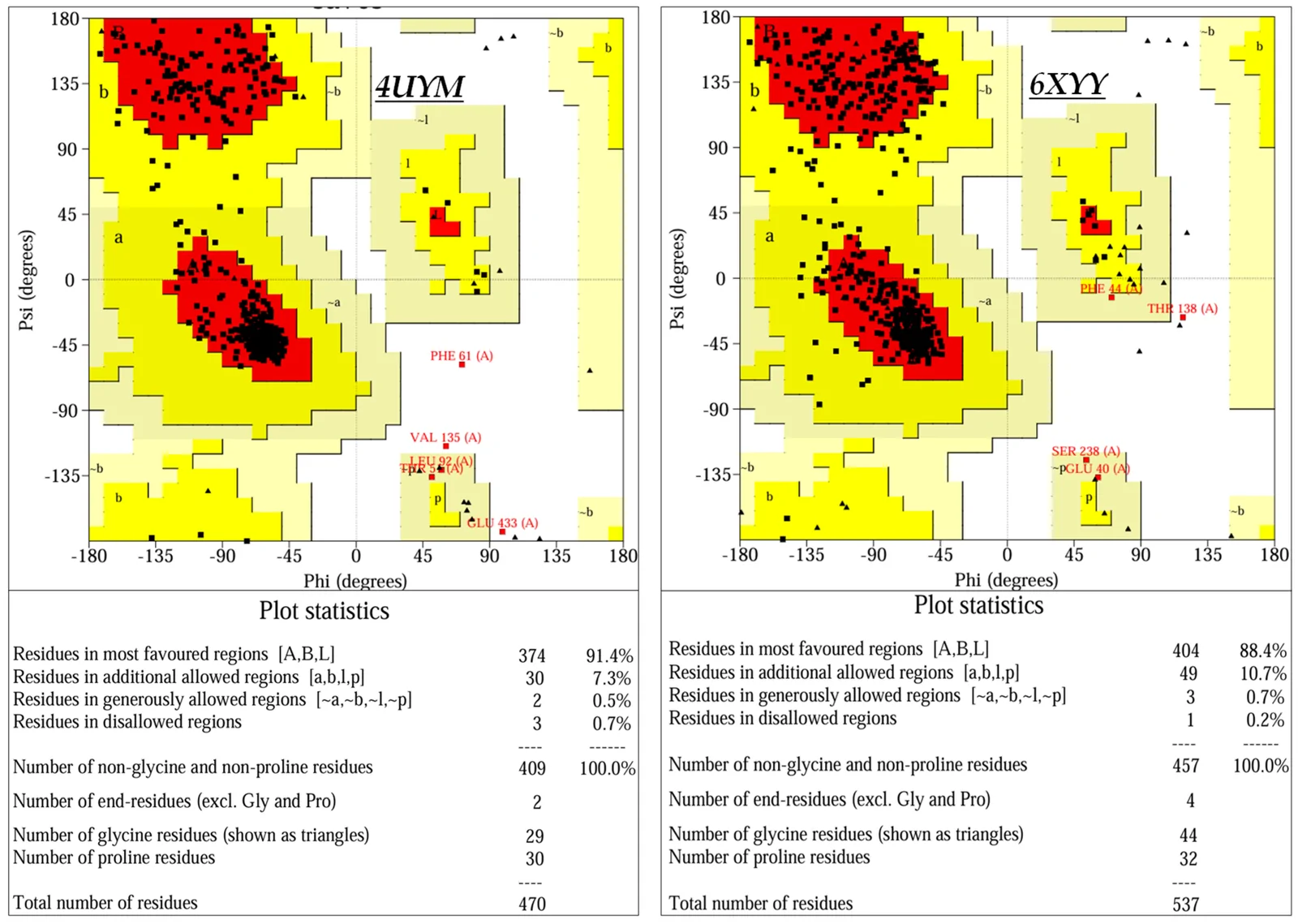
Ramachandran plot-based stereochemical assessment of the receptors used for docking. (**Left**) *Aspergillus fumigatus* CYP51B (PDB ID: 4UYM); (**Right**) *Drosophila melanogaster* acetylcholinesterase (PDB ID: 6XYY).

Before ligand ranking was interpreted, the global stereochemical quality of both receptors was examined using the Ramachandran plots in Figure 7. For 4UYM, 374 of 409 non-glycine/non-proline residues (91.4%) were located in the most favored regions, 30 residues (7.3%) in additionally allowed regions, and two residues (0.5%) in generously allowed regions; only three residues (0.7%) occurred in disallowed regions. For 6XYY, 404 of 457 non-glycine/non-proline residues (88.4%) occupied the most favored regions, 49 (10.7%) the additionally allowed regions, three (0.7%) the generously allowed regions, and one (0.2%) a disallowed region. Thus, approximately 99.2% and 99.8% of the evaluated residues in 4UYM and 6XYY, respectively, fall within accepted conformational space. The low outlier fractions support the overall stereochemical suitability of the structures for docking, while also recognizing that a global Ramachandran assessment cannot by itself exclude local uncertainty within an individual binding-site loop [37].

Table 3 shows a target-dependent but chemically coherent ranking. Against CYP51B, alpha-thujene was the highest-ranked essential-oil constituent (−6.1 kcal/mol), followed closely by p-cymene (−6.0 kcal/mol), whereas voriconazole reached −7.2 kcal/mol. Alpha-thujene therefore remained 1.1 kcal/mol less favorable than the reference, and the narrow 0.8 kcal/mol span across the eight constituents indicates that no single component overwhelmingly dominated predicted CYP51B recognition. Against AChE, p-cymene ranked first (−6.9 kcal/mol), followed by isopulegol (−6.3 kcal/mol), while tacrine derivative 760 scored −10.5 kcal/mol. The 3.6 kcal/mol gap to the reference and the small size of p-cymene are consistent with partial rather than complete exploitation of the AChE gorge. These values support prioritization, not equivalence to the reference inhibitors, because Vina scores are approximate ranking functions rather than experimental binding free energies. Notably, Santolina triene, the dominant GC-MS constituent of the oil (48.44%), produced only intermediate scores (−5.5 kcal/mol for 4UYM and −5.7 kcal/mol for 6XYY). The biological activity of the whole oil therefore cannot be explained by abundance alone and may reflect additive or synergistic contributions from several constituents, differences in volatility and exposure, or targets not represented in the present docking panel.

**Figure 8:**
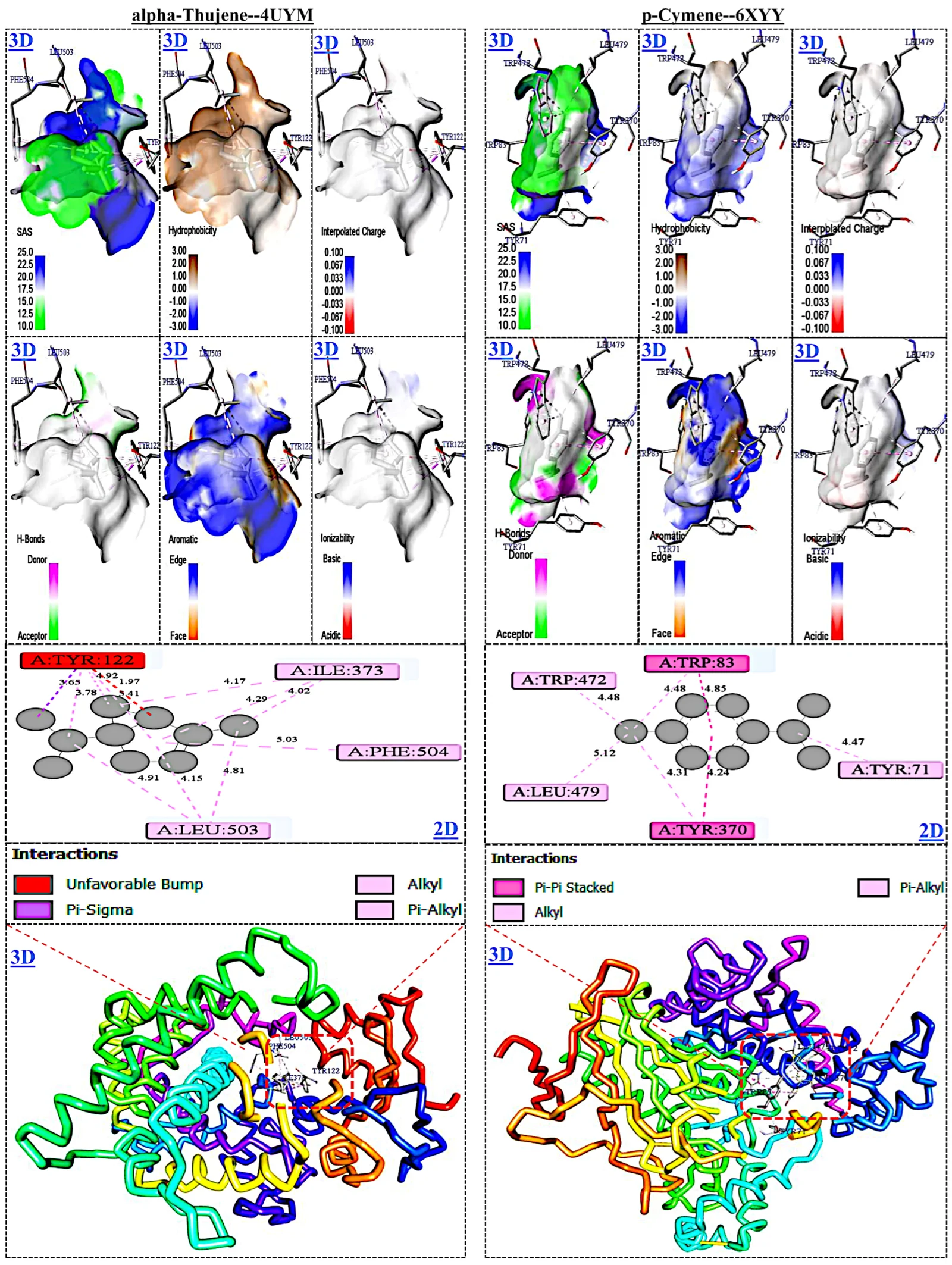
Integrated 2D and 3D binding-mode analysis of the highest-ranked essential-oil constituents: alpha-thujene in CYP51B (4UYM) and p-cymene in acetylcholinesterase (6XYY). The panels include solvent-accessible surface (SAS), hydrophobicity, interpolated charge, hydrogen-bond donor/acceptor character, aromaticity, ionizability, 2D interaction maps, and global protein views. Residue labels refer to chain A, and annotated contact distances are expressed in Å.

The highest-ranked poses were examined through complementary 2D and 3D representations in Figure 8. Alpha-thujene occupies a predominantly nonpolar CYP51B pocket bordered by Tyr122, Ile373, Leu503, and Phe504. Its compact hydrocarbon scaffold is supported mainly by alkyl, pi-alkyl, and pi-sigma contacts, while the solvent-accessible-surface and hydrophobicity maps show close shape and lipophilic complementarity [38,39,40]. The charge, hydrogen-bond, and ionizability surfaces contribute little, consistent with the absence of heteroatoms and hydrogen-bond donors or acceptors. A localized unfavorable contact with Tyr122 (1.97 Å in Figure 8) indicates residual steric strain; together with the absence of polar or metal-coordinating functionality, this suggests a structural rationale for the moderate −6.1 kcal/mol score and its weaker affinity ranking relative to voriconazole. In 6XYY, the aromatic ring of p-cymene is accommodated within a hydrophobic and aromatic region and forms pi-pi stacking with Trp83 and Tyr370, supplemented by pi-alkyl or alkyl contacts with Trp472, Tyr71, and Leu479 [41,42]. This interaction pattern explains why p-cymene performs particularly well at AChE: its aromatic face can engage the gorge through directional pi contacts, while its methyl and isopropyl substituents reinforce nonpolar packing. Nevertheless, the absence of hydrogen bonds, ionic anchoring, and a broader polar-contact network limit the interaction pattern relative to the tacrine derivative. The maps therefore support geometric plausibility and local complementarity of the selected poses, but they do not constitute proof of dynamic stability; molecular-dynamics simulations and direct enzyme-inhibition assays would be required to establish persistence and functional inhibition. No per-residue energy decomposition was performed; the contact analysis describes interaction geometry rather than energetic contributions. The 0.1 kcal/mol separation between alpha-thujene and p-cymene at CYP51B does not establish a robust affinity difference.

### 3.5. ADMET Prediction

Early ADMET profiling was used to complement target-based prioritization with an exposure- and safety-oriented perspective. For small, volatile, and hydrophobic essential-oil constituents, a favorable docking pose does not ensure aqueous dispersibility, appropriate biological exposure, or acceptable toxicological selectivity [43,44]. The admetSAR3.0 outputs were therefore examined to identify physicochemical drivers, potential transport and metabolic liabilities, and model-derived human-health alerts before experimental follow-up.

**Figure 9:**
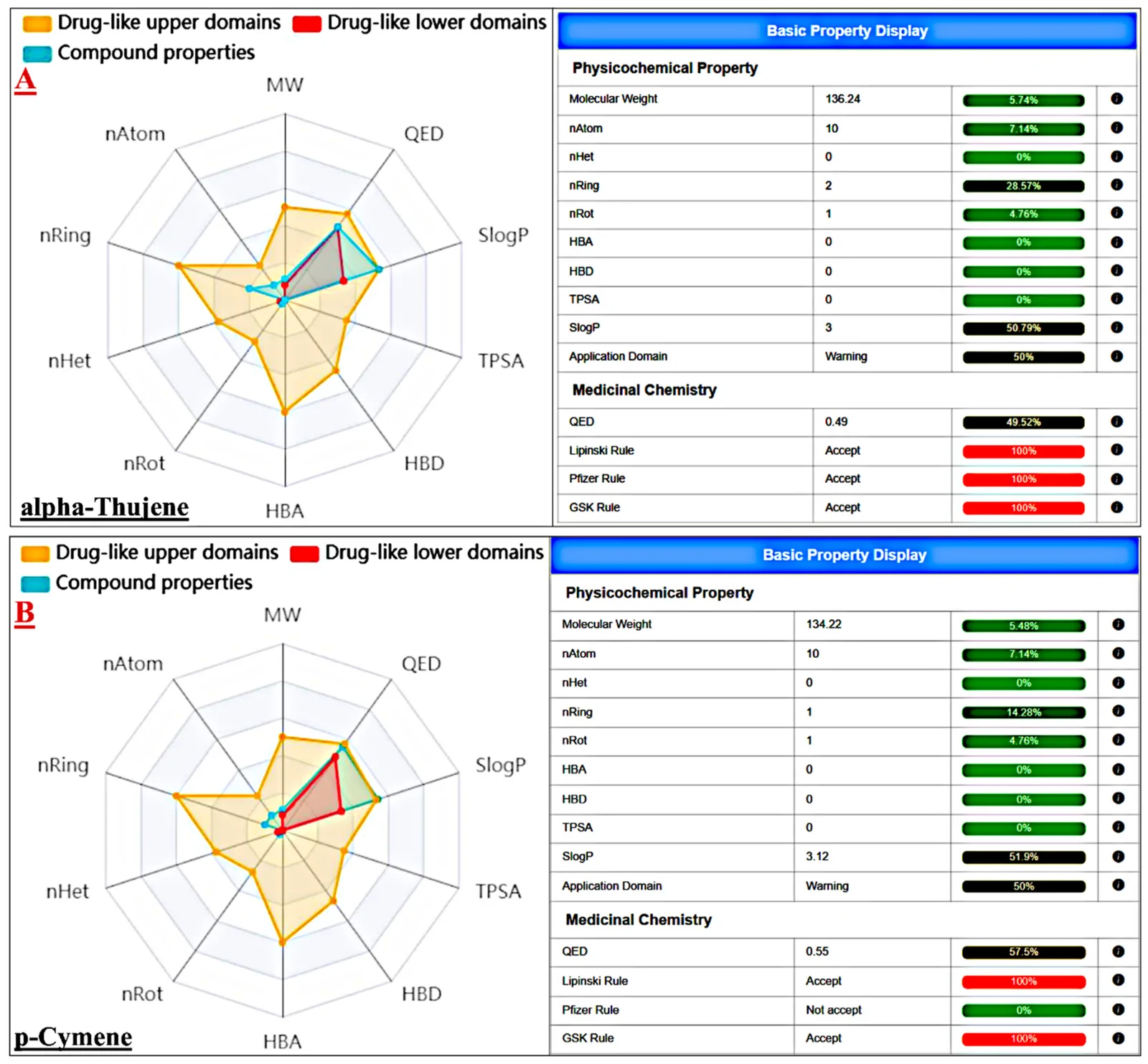
admetSAR3.0 physicochemical and medicinal-chemistry profiles of (**A**) alpha-thujene and (**B**) p-cymene. Radar plots show compound properties relative to rule-based upper and lower domains, whereas the accompanying tables summarize descriptors, applicability-domain status, QED, and medicinal-chemistry filters.

Figure 9 reveals closely related, highly lipophilic profiles. Alpha-thujene (molecular weight 136.24) and p-cymene (molecular weight 134.22) each contain 10 heavy atoms, no heteroatoms, one rotatable bond, zero hydrogen-bond donors, zero hydrogen-bond acceptors, and a topological polar surface area of 0 Å^2^; alpha-thujene contains two rings, whereas p-cymene contains one. Their SlogP values are 3.00 and 3.12, respectively, indicating a strong preference for nonpolar environments and providing a physicochemical basis for the hydrophobic complementarity observed in Figure 8 [45]. At the same time, the absence of polar surface area predicts limited aqueous solvation and suggests that experimentally observed activity may be strongly formulation- and exposure-dependent. The QED values are moderate (0.49 for alpha-thujene and 0.55 for p-cymene). Both compounds pass the Lipinski and GSK filters, whereas p-cymene fails the Pfizer filter while alpha-thujene passes. These rule-based outputs should not be equated with efficacy or safety, especially because both molecules are reported with an “Application Domain: Warning”. This warning indicates that at least part of the prediction lies near or outside the model’s best-supported chemical space and that downstream interpretation should retain explicit uncertainty.

**Figure 10:**
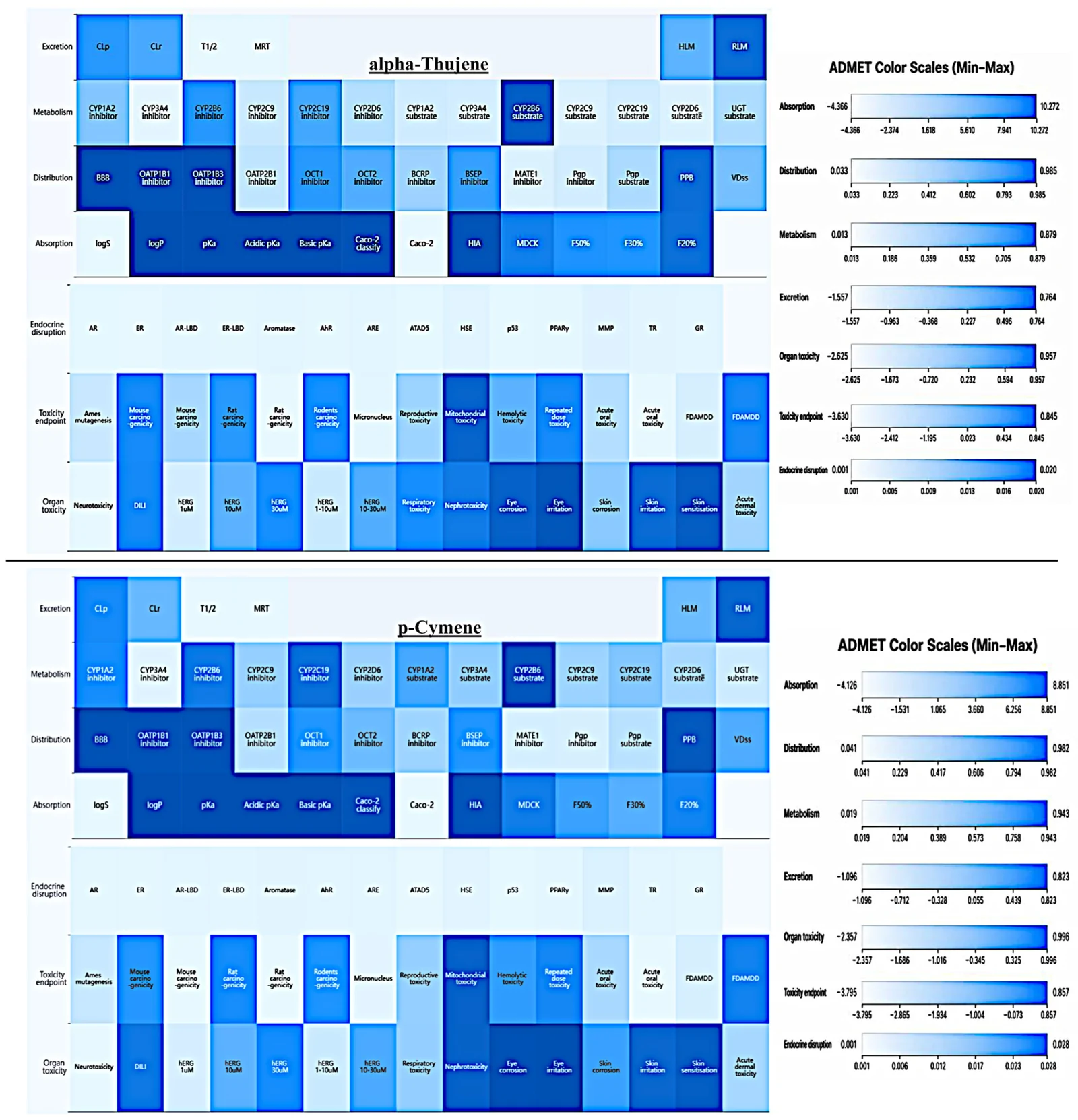
Min–max normalized admetSAR3.0 profiles for alpha-thujene (**upper panels**) and p-cymene (**lower panels**) across absorption, distribution, metabolism, excretion, organ toxicity, toxicity endpoints, and endocrine disruption. Darker blue denotes a higher model output within the corresponding category; because endpoint scales and meanings differ, colors should not be compared as universal probabilities across categories.

Figure 10 extends the physicochemical picture across a broad set of ADME and toxicity endpoints. The absorption and distribution panels of both compounds show a pattern dominated by strong logP, blood-brain-barrier, and plasma-protein-binding outputs, whereas logS is among the lowest-valued descriptors. This profile is consistent with small neutral hydrocarbons that preferentially partition into lipid phases but have limited aqueous solubility. P-glycoprotein inhibition and substrate outputs are comparatively weak, while OATP1B1/OATP1B3 and selected OCT/BSEP transporter outputs are more prominent; these should be regarded as hypotheses for possible transporter interactions rather than established liabilities. The metabolic panels show the strongest relative outputs around CYP2B6 substrate/inhibition and CYP2C19 inhibition, with p-cymene also displaying a comparatively prominent CYP1A2-inhibition signal, whereas CYP3A4-inhibition outputs remain weak. The excretion panels show stronger rat- than human-liver-microsome outputs and comparatively low half-life and mean residence-time signals, although kinetic interpretation requires the original endpoint definitions and numerical values. Within the human-health panels, the most conspicuous model-derived alerts for both compounds involve drug-induced liver injury, nephrotoxicity, eye corrosion/irritation, skin irritation/sensitization, mitochondrial toxicity, repeated-dose toxicity, and selected rodent-carcinogenicity classifiers. By contrast, neurotoxicity, the most stringent hERG category, and the endocrine-disruption endpoints are generally less prominent. The slightly darker skin- and eye-related outputs for p-cymene suggest a potentially less favorable local-tolerance profile, but the difference should not be overinterpreted. Because the heat maps are min-max normalized by broad category and combine continuous descriptors with classification scores, direct quantitative comparison between unrelated cells is not valid. These outputs are computational alerts, not confirmed toxicities. Taken together, Figure 9 and Figure 10 expose a central trade-off: the hydrophobicity that improves complementarity within CYP51B and AChE may simultaneously reduce solubility and increase exposure-related liabilities. Alpha-thujene and p-cymene therefore remain rational candidates for targeted antifungal and insecticidal validation, but their prioritization should be conditioned on formulation studies, direct enzyme assays, cytotoxicity, irritation and sensitization testing, and environmental-safety evaluation rather than docking score alone.

## 4. Conclusions

This study demonstrates the antifungal and insecticidal potential of *Santolina chamaecyparissus* L. essential oil and highlights the influence of concentration, exposure route, and exposure duration on its biological efficacy. The integration of GC–MS profiling with molecular docking suggests that biological activity cannot be attributed solely to the most abundant constituent, as α-thujene and p-cymene showed the most favorable predicted interactions with CYP51B and AChE, respectively. Nevertheless, these computational results represent mechanistic hypotheses and require experimental confirmation. In particular, future studies should evaluate the antifungal and insecticidal activities of prioritized pure constituents, individually and in defined mixtures, and directly assess their effects on the proposed molecular targets. Additional toxicological and environmental-safety investigations will also be necessary before practical applications can be considered.

## Figures and Tables

**Table 1:** Volatile constituents tentatively identified in *Santolina chamaecyparissus* L. essential oil by GC–MS based on mass-spectral library matching.

Peak	Proposed Compound	RT (min)	Area (%)	Molecular Formula	Chemical Class	Match	R. Match
1	Cyclofenchene	7.72	0.32	*C_10_H_16_*	Monoterpene hydrocarbon	800	817
2	Camphene	9.03	1.04	*C_10_H_16_*	Monoterpene hydrocarbon	844	864
3	α-Phellandrene	9.94	2.17	*C_10_H_16_*	Monoterpene hydrocarbon	831	844
4	β-Pinene	10.03	2.39	*C_10_H_16_*	Monoterpene hydrocarbon	945	952
5	β-Myrcene	10.63	5.17	*C_10_H_16_*	Monoterpene hydrocarbon	903	916
6	p-Cymene	11.80	1.60	*C_10_H_14_*	Monoterpene hydrocarbon	835	835
7	α-Pinene	11.96	14.05	*C_10_H_16_*	Monoterpene hydrocarbon	914	918
8	Santolina triene	13.25	48.44	*C_10_H_16_*	Monoterpene hydrocarbon	819	825
9	Camphor	16.13	8.80	*C_10_H_16_O*	Oxygenated monoterpene	928	932
10	Isopulegol	17.37	1.28	*C_10_H_18_O*	Oxygenated monoterpene	875	875
11	α-Thujene	17.65	4.26	*C_10_H_16_*	Monoterpene hydrocarbon	879	883
12	p-Cymen-8-ol	21.38	0.58	*C_10_H_14_O*	Oxygenated monoterpene	779	792
13	Carveol	22.42	0.29	*C_10_H_16_O*	Oxygenated monoterpene	744	796
14	Isoborneol	33.23	1.03	*C_10_H_18_O*	Oxygenated monoterpene	644	695

**Table 2:** Minimum inhibitory concentration (MIC) and minimum fungicidal concentration (MFC) of *Santolina chamaecyparissus* L. essential oil against the tested fungal strains.

	*Aspergillus brasiliensis*		*Fusarium oxysporum*	
	MIC (mg/mL)	MFC (mg/mL)	MIC (mg/mL)	MFC (mg/mL)
*Santolina chamaecyparissus EO*	4	4	4	4

**Table 3:** Molecular docking performance of *Santolina chamaecyparissus* essential-oil constituents against antifungal and insecticidal molecular targets.

Molecular Target	PDB ID	Ligand/Compound	Best Docking Score (kcal/mol)	Rank Within EO Constituents	Delta vs. Reference (kcal/mol)
**Sterol 14-alpha-demethylase** **(CYP51B)** *Aspergillus fumigatus*	**4UYM**	Voriconazole	−7.2	Ref.	-
		alpha-Thujene	−6.1	1	+1.1
		p-Cymene	−6.0	2	+1.2
		Camphene	−5.7	3	+1.5
		Camphor	−5.6	4	+1.6
		Isoborneol	−5.5	5	+1.7
		Isopulegol	−5.5	6	+1.7
		Santolina triene	−5.5	7	+1.7
		Myrcene	−5.3	8	+1.9
**Acetylcholinesterase (AChE)** *Drosophila melanogaster*	**6XYY**	Tacrine derivative 760	−10.5	Ref.	-
		p-Cymene	−6.9	1	+3.6
		Isopulegol	−6.3	2	+4.2
		alpha-Thujene	−6.0	3	+4.5
		Myrcene	−5.9	4	+4.6
		Camphor	−5.7	5	+4.8
		Santolina triene	−5.7	6	+4.8
		Camphene	−5.3	7	+5.2
		Isoborneol	−5.2	8	+5.3

## Data Availability

All data supporting the findings of this study, including the relevant computational files and associated datasets, are freely and publicly available in the GitHub repository: https://github.com/khibech/Santolina-chamaecyparissus-L. (first deposited on 4 September 2026; accessed on 24 September 2026). The repository can be accessed without restriction and contains the data required to reproduce and further analyze the results reported in this work.
